# Liquid Biopsy: Current Status and Future Perspectives

**DOI:** 10.3390/cancers15123205

**Published:** 2023-06-15

**Authors:** Vesselin Baev, Danijela Koppers-Lalic, Bruno Costa-Silva

**Affiliations:** 1 Department of Plant Physiology and Molecular Biology, University of Plovdiv, 4000 Plovdiv, Bulgaria; baev@uni-plovdiv.bg; 2 Mathematical Institute, Leiden University, 2333 CA Leiden, The Netherlands; 3 Champalimaud Physiology and Cancer Programme, Champalimaud Foundation, 1400-038 Lisbon, Portugal

Since the discovery of the Bence Jones protein in the middle to late 1800s and the subsequent identification of the carcinoembryonic antigen and alpha-fetoprotein in the 1970s, it has been demonstrated that the analysis of biofluids is essential to the diagnostic and follow-up processes of cancer [1]. Although proteins are still the most common cancer biomarkers, other molecules and structures, such as circulating cell-free tumor DNA (cfDNA); micro (microRNA), messenger (mRNA), and non-coding long and small RNAs; circulating tumor cells (CTCs); tumor-educated platelets (TEPs); and extracellular vesicles (EVs), have emerged as promising sources of cancer biomarkers [2]. In the same manner as traditional tissue biopsies, several of these new sources of markers provide essential molecular information on cancer cells. This information includes gene mutations and gene expression profiles. As a result, over the course of the past 13 years, we have come to refer to them as “liquid biopsies” [3].

Significant progress has been made in the development of methods for extracting cancer-related products from biofluids and linking the contents of these extracts to the diagnosis, prognosis, and treatment response of tumor lesions. This is particularly the case for liquid biopsies that are based on CTC and cfDNA [2]. However, additional progress in establishing optimal and uniform technical procedures for isolating and analyzing biomolecules linked to cancer and identifying improved disease-specific biomarker panels is still required before liquid biopsies can be adopted in clinical practice.

This Special Issue concerns the use of cancer liquid biopsies as emerging diagnostic strategies for application to both adult and pediatric tumors. Our goal is to provide up-to-date information on cancer liquid biopsies and offer pertinent information on emerging technologies that can be used to obtain cancer materials in a non-invasive manner and profile their molecular compositions.

The use of liquid biopsies (more specifically, circulating DNA) to assess pediatric tumors was systematically reviewed by Greuter et al. They demonstrated that liquid biopsies can be used to identify and follow the progression of disease, particularly in high-grade gliomas and medulloblastomas [4]. On the same topic, Manukonda et al. mapped the microRNA, mRNA, and long non-coding RNA content of plasma EVs derived from patients with retinoblastoma. They discuss their possible relationship with the epigenetic regulation of the cell cycle, metabolism, and tumor-associated signaling pathways [5].

This Special Issue also showcases new technologies that improve the use of liquid biopsies as a diagnostic tool for cancer. This includes the work by Arthur et al., who demonstrated that multiplexed droplet digital PCR for the study of cfDNA in cerebrospinal fluid can be utilized as a very sensitive method for the diagnosis of medulloblastoma [6]. In addition, Telekes et al. reviewed the progress in the potential use of cfDNA, both by itself and in combination with other liquid biopsy modalities, to detect multiple types of primary and metastatic cancer lesions and longitudinally monitor their responses to therapy [7]. In a different study, Diest and colleagues examined the value of nipple aspirate fluid as a source of valuable cancer biomolecules (such as microRNAs) for use in breast cancer liquid biopsy [8]. Additionally, Glogovitis et al. demonstrated the application of a framework that is based on Galaxy [9]. miRGalaxy is an open-source platform for analyzing NGS data, focusing on microRNAs and their sequence variants—isomiRs (template and non-template)—across samples. The miRNA and isomiR-based cancer research field has witnessed a remarkable evolution over the last few years, and the miRGalaxy platform can be successfully used in the process of discovering relevant cancer biomarkers in biofluids.

In addition, a comprehensive study conducted by Wen and colleagues shows that bronchial lavage can be used as a source of methylated tumor cfDNA for the diagnosis of lung cancer. This helps overcome the low sensitivity of cfDNA profiling in the plasma of these patients [10]. This strategy is investigated further by Kim et al., who show that EVs isolated from bronchial lavage can also be used to perform EGFR genotyping in advanced lung cancer patients [11].

The practical aspects related to the potential use of liquid biopsies, especially those involving circulating tumor DNA, for the early detection of breast cancer were evaluated by van der Poort et al., who utilized a microsimulation model to assess the potential benefits, harms, and costs of this diagnosis strategy [12]. Another perspective, involving the use of liquid biopsies for the diagnosis of oral cancer, was reviewed by Adeola et al., who discuss the relevant factors before, during, and after sample collection with respect to the utilization of different liquid biopsy modalities in resource-limited settings, namely, countries on the African continent [13].

We expect that this Special Issue will contribute to the development of liquid biopsies as improved tools not only for the diagnosis and follow-up of cancer but also as a still underexplored source of specific biomarkers necessary for the development of patient-tailored precision medicine.
